# Surveillance Colonoscopy Revealing Asymptomatic Low-Grade Appendiceal Mucinous Neoplasm

**DOI:** 10.7759/cureus.16222

**Published:** 2021-07-06

**Authors:** Jagmeet S Grewal, Elliot Berger, Jacob Garner, Savannah L Mayer, Jennifer S Beaty

**Affiliations:** 1 Medicine, Des Moines University School of Osteopathic Medicine, Des Moines, USA; 2 Surgery, Des Moines University School of Osteopathic Medicine, Des Moines, USA

**Keywords:** low grade appendiceal mucinous neoplasm, surveillance colonoscopy, neoplasm, appendix, adenocarcinoma of the appendix

## Abstract

Appendicular mucinous neoplasms are a collection of rare tumors with diverse clinical presentations and pathologic potential, which can cause diagnostic and therapeutic challenges. Traditionally, they are diagnosed by radiologic imaging or identified intraoperatively; however, rarely, they may be diagnosed during an endoscopic procedure. In this unusual case, we present the case of a 62-year-old Caucasian male undergoing routine surveillance colonoscopy due to a history of colonic neoplasia. During the colonoscopy, a submucosal, non-bleeding 1cm mass of benign appearance was found in the appendix. Further workup determined the mass was likely a mucocele, and surgical consultation was recommended. The patient denied any symptoms suggestive of a mucinous neoplasm prior to and during evaluation. A laparoscopic appendectomy was subsequently performed, and the histopathology report confirmed the diagnosis of a low-grade appendiceal mucinous neoplasm. The patient recovered without complications and continued to deny any symptoms during his postoperative course and follow-up care. Given their rare incidence and unpredictable nature, appendiceal mucinous neoplasms remain difficult to identify. Discovering a low-grade mucinous neoplasm in an asymptomatic patient via colonoscopy illustrates the spectrum of unique presentations and modalities for diagnosis.

## Introduction

Appendiceal mucinous neoplasms are a group of tumors that are relatively rare and can present in a variety of clinical and pathological presentations. These neoplasms can have either benign or malignant activity, and classification can be controversial [1]. Appendiceal mucinous neoplasms account for less than 0.5% of gastrointestinal tumors [2]. Due to the unpredictable nature of their clinical courses, discovering them can take place in many different ways. In this article, we will discuss a case of low-grade appendicular mucinous neoplasm found on surveillance colonoscopy.

## Case presentation

**Figure 1:**
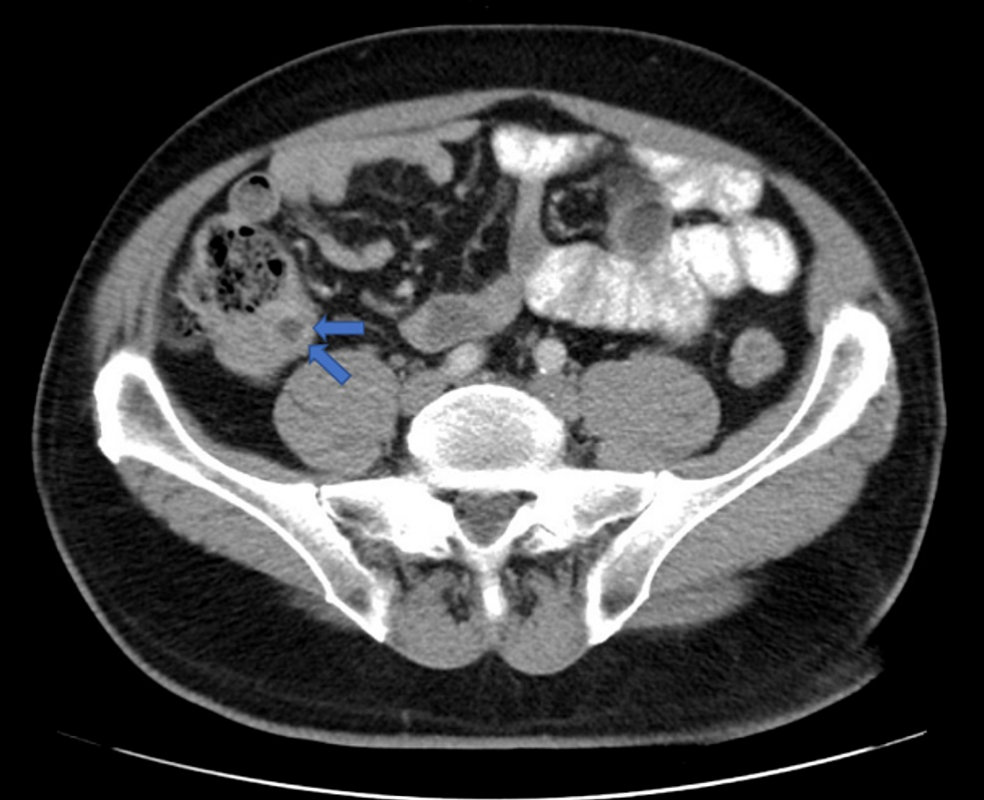
Abdominal transverse view CT scan with contrast demonstrating focal hypodense mass within the base of the appendix, which measures 1cm x 1cm. The more distal portions of the appendix are unremarkable. There is no evidence of periappendiceal fat stranding. CT: Computerized tomography

**Figure 2:**
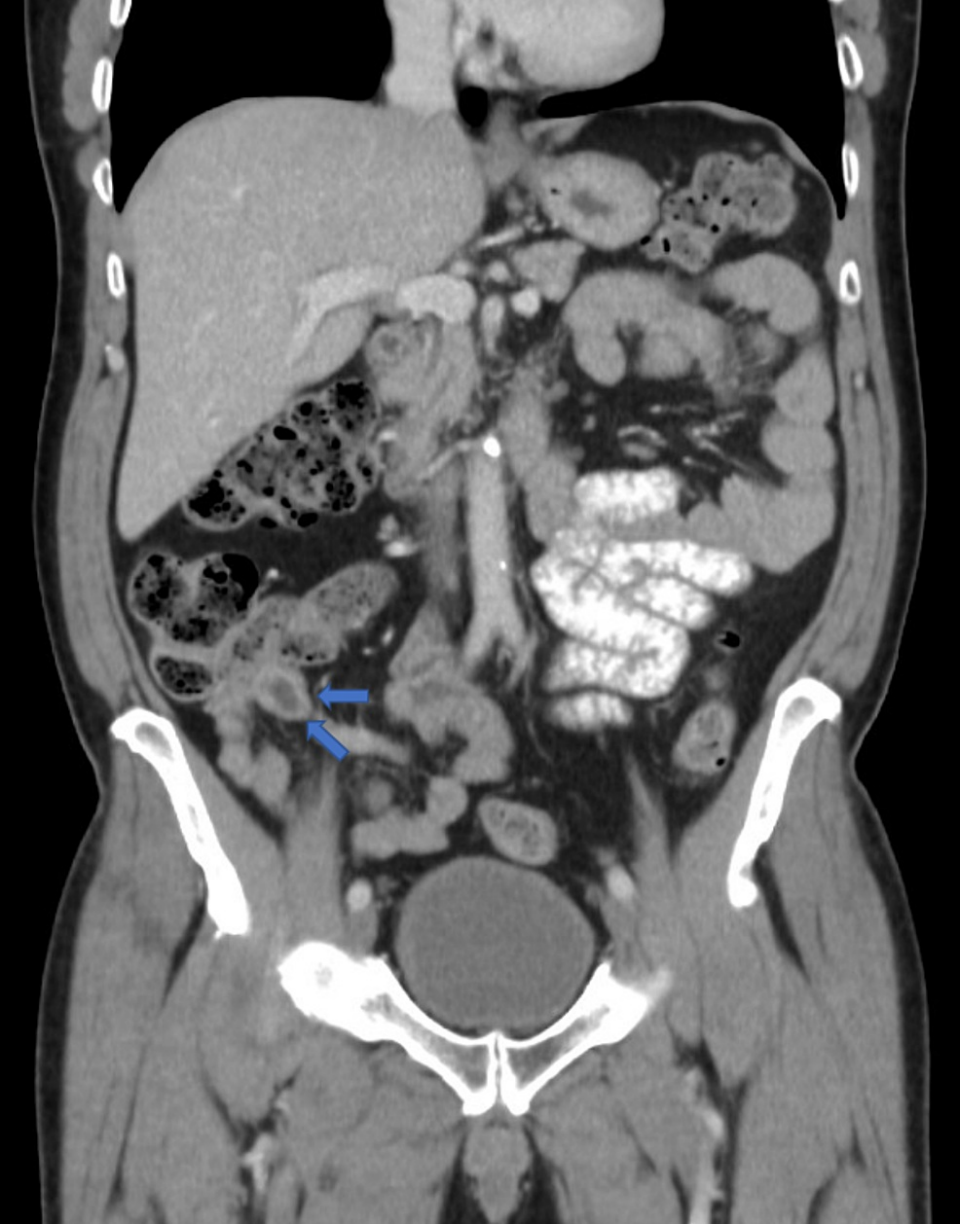
Abdominal coronal view CT scan with contrast demonstrating focal hypodense mass within the base of the appendix, which measures 1cm x 1cm. The more distal portions of the appendix are unremarkable. There is no evidence of periappendiceal fat stranding. CT: Computerized tomography

A 62-year-old Caucasian male with a past medical history of colonic neoplasia was undergoing surveillance colonoscopy when an appendiceal mass was identified. A submucosal non-hemorrhaging 1cm mass of benign appearance was found in the appendix. No other abnormalities were identified during the surveillance colonoscopy. Follow-up imaging was ordered, and an abdominal CT scan with contrast demonstrated a focal hypodense mass at the mid appendix, which measured 1cm x 1cm. The more distal portions of the appendix were unremarkable, and there was no evidence of periappendiceal fat stranding (Figures 1, 2).

Prior to colonoscopy, the patient denied any symptoms, including unexplained weight loss, abdominal pain, nausea, vomiting, fever, or chills. History and physical examination were unremarkable. Pre-surgical blood work was within normal limits. Surgical consultation one month later recommended laparoscopic appendectomy with the possibility of open appendectomy depending on the difficulty of removing the mass and/or follow-up right hemicolectomy depending on the extent of invasion on the pathological report.

Surgery occurred shortly after the surgical consultation. The appendix was identified, and the mass did not appear to involve the appendiceal base. Firmness was appreciated to the mid appendix. The appendix was removed. The specimen was palpated in the endocatch bag, and there appeared to be a mass of the mid appendix with the base of the appendix feeling normal. The specimen was sent to pathology.

**Figure 3:**
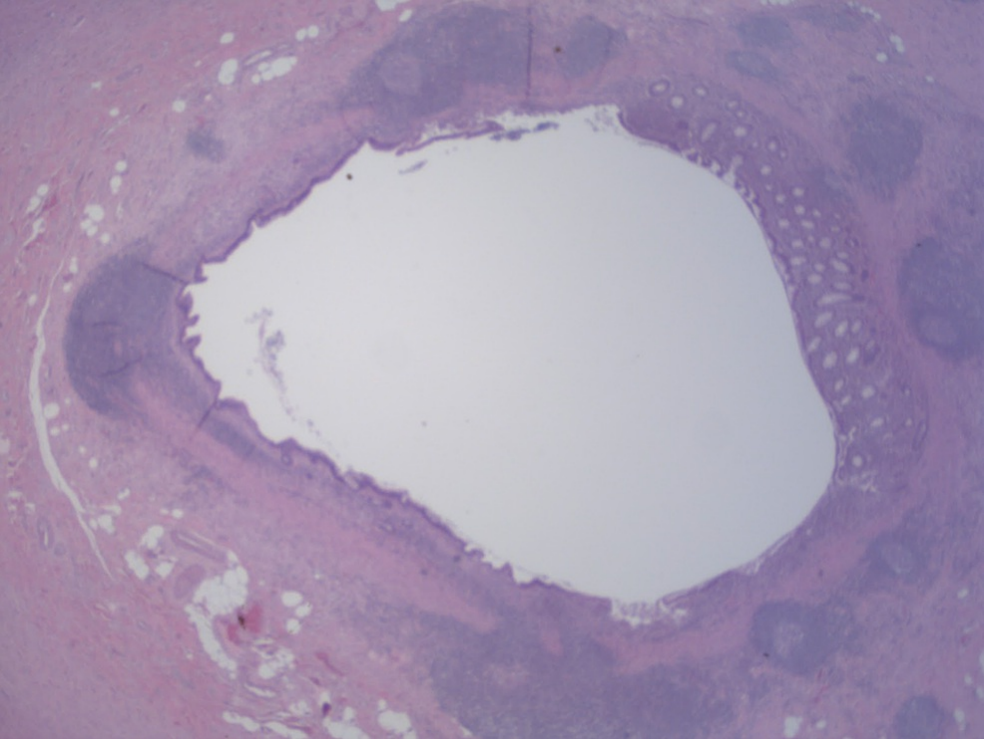
Whole section of the appendix body with a dilated lumen. Normal mucosa is seen on the right with low-grade adenomatous-type epithelium on the left. (H&E, 20X) (H&E, 20X): Hematoxylin and eosin stain, at 20 times magnification

**Figure 4:**
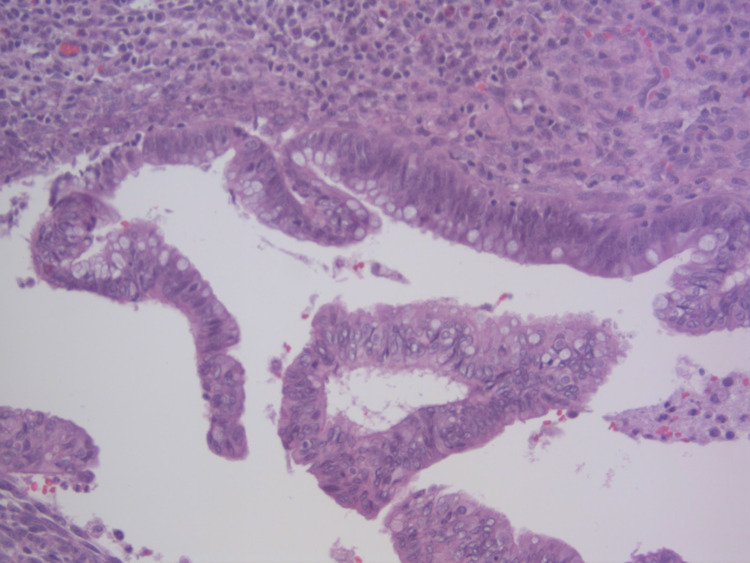
High power view of adenomatous type epithelium with pseudostratified nuclei and decreased mucin production. (H&E, 200X) (H&E, 200X): Hematoxylin and eosin stain, at 200 times magnification

The histopathology report demonstrated a low-grade appendiceal mucinous neoplasm, non-invasive, with intramural rupture and chronic abscess (Figures 3, 4). The surgical margin was negative for involvement. The patient has continued to be symptom-free following surgery and was referred to hematology and oncology for further follow-up. Repeat colonoscopy in one year was also recommended.

## Discussion

While appendiceal tumors comprise only a small percentage of gastrointestinal neoplasms, the sub-classification of low-grade appendiceal mucinous neoplasm (LAMN) is even rarer by comparison. LAMNs are three times more likely to affect females than males and are typically found in patients between the ages of 50-60 years [3,4]. The clinical presentation of an appendiceal neoplasm varies greatly for both men and women, making it difficult to diagnose on initial presentation. Some patients remain asymptomatic, while others present with weight loss, hematochezia, bowel obstruction, or bowel perforation, which may be signs of advanced disease. Because of this spectrum of possible presentations, LAMNs result in broad differentials and can be mistaken for various pathologies such as appendiceal diverticulitis or endometriosis [4,5]. It is also important to note that nearly a quarter of patients present without symptoms, and therefore are diagnosed with a LAMN based on an incidental finding [5]. In general, the most common presentation that leads to a diagnosis of LAMN is right lower quadrant abdominal pain in the setting of acute appendicitis [6].

There is debate over the origin and classification of LAMNs; however, one popular theory is that these neoplasms arise from mucoceles in the appendix. A mucocele is a cyst-like space that harbors mucin-producing cells, which progress by filling the appendix with mucus [3]. In response to this mucus accumulation, the appendix begins to dilate, which can cause ulceration and loss of epithelium with an increased risk of rupture [4,5]. Mucoceles are classified into histological groups of both non-neoplastic and neoplastic origins. Non-neoplastic mucoceles are typically caused by hyperplasia of the mucosa or retention cysts secondary to luminal obstruction [3]. Conversely, neoplastic mucoceles are more complicated to classify and are often compared to tumors of the small and large intestines based on similar molecular pathways that lead to precursor lesions [4]. The World Health Organization divides these neoplasms into three categories: mucinous adenoma, LAMN, and appendiceal adenocarcinoma [6]. Continued research has led to additional classifications of cystadenomas as well as high-grade appendiceal mucinous neoplasms (HAMNs) [7].

Multiple modalities can be used to initially identify appendiceal mucinous neoplasms, including ultrasound, CT, and MRI. Some favor using ultrasound in order to differentiate between acute appendicitis and LAMNs, as it allows for the visualization of mucinous effusion and the characteristic appearance of an “onion skin” mucocele [8,9]. However, CT imaging is considered a superior modality and is more commonly used to establish a working diagnosis [6,8]. Using CT imaging also minimizes misdiagnosis of an adnexal mass as compared to using ultrasound. Findings supportive of a mucinous neoplasm include an appendiceal lumen that is greater than 13mm in addition to visualizing a calcified cystic formation [9]. Once identified on imaging, surgical resection with histopathologic examination is indicated to confirm the diagnosis of LAMNs. Epithelial cells from biopsied tissue show a flattened and villiform growth pattern with effacement of lamina propria and obliterated muscularis mucosae [10,11]. The cytoplasm of this neoplastic tissue appears abundant in mucin and is defined by the capacity to invade the appendiceal wall and the potential to spread into the peritoneum. If peritoneal seeding occurs, neoplastic cells may extrude mucin into the peritoneal cavity [3].

Surgical resection is the standard treatment of low-grade appendiceal mucinous neoplasms as it is the only management that is potentially curative. Appropriate surgical management is critical because rupture of the neoplasm in the appendix allows for mucinous neoplastic cells to spread throughout the peritoneum [9]. LAMNs that are confined to the appendix and lack malignant potential are typically treated with a single appendectomy. If the unruptured LAMN cannot be safely resected laparoscopically, conversion to an open operation is recommended [11]. Some literature recommends right-sided hemicolectomy as the mainstay of therapy, but there lacks sufficient evidence that this procedure results in a superior prognosis [12]. Guaglio et al. examined 41 patients post-appendectomy (n=31) or right colectomy (n =5) with close biochemical and radiographic surveillance. An appendiceal rupture was present in 21 patients. The 5-year recurrence-free survival was 95%, with only two patients progressing to peritoneal recurrences at 18 and 22 months post-appendectomy [13].

The rate of metastatic disease from appendiceal adenocarcinoma to regional lymph nodes ranges from 20% to 67%, which means nonmetastatic adenocarcinoma confined to the appendix should be treated with a right hemicolectomy [13]. Furthermore, this would allow for improved staging and may have a therapeutic benefit. If there are peritoneal metastases, routine right hemicolectomy to remove clinically normal lymph nodes is not recommended; several single and retrospective studies have failed to demonstrate a survival benefit to right colectomy vs. appendectomy along with patients undergoing cytoreductive surgery (CRS) and chemotherapy [14]. For patients with appendiceal neoplasms with peritoneal metastases, surgery remains the standard therapy. The aim of CRS is the complete removal of gross disease and is often combined with chemotherapy for best results. The overall procedure of CRS includes selective peritonectomies, specifically over the diaphragms and within the pelvis, removal of tumors on the surfaces of the small intestine and colon, supracolic omentectomy, and other resections as indicated by involvement such as splenectomy [15].

There are no formal surveillance guidelines for appendiceal neoplasms after appendectomy. It is suggested to obtain MRI with tumor markers, carcinoembryonic antigen (CEA)/carbohydrate antigen 19-9 (CA 19-9)/cancer antigen 125 (CA 125), every six months for two years because most early recurrences occur within that time frame. Patients with HAMNs or who have had a right hemicolectomy should undergo CT or MRI every four-six months for the first two years and yearly thereafter for greater than five years [16].

## Conclusions

A low-grade appendiceal mucinous neoplasm is treated with surgical resection through an appendectomy for unruptured tumors localized to the appendix and not involving the appendiceal base or right hemicolectomy, which has the added advantage of allowing the histopathologist to ascertain the status of lymph node metastasis. In cases of peritoneal metastasis or a ruptured appendix, a cytoreductive surgery combined with intraperitoneal heated chemotherapy is the recommended approach nowadays.
